# A brain basis for musical hallucinations^☆^

**DOI:** 10.1016/j.cortex.2013.12.002

**Published:** 2014-03

**Authors:** Sukhbinder Kumar, William Sedley, Gareth R. Barnes, Sundeep Teki, Karl J. Friston, Timothy D. Griffiths

**Affiliations:** a Institute of Neuroscience, Medical School, Newcastle University, Newcastle upon Tyne, UK; b Wellcome Trust Centre for Neuroimaging, London, UK

**Keywords:** Musical hallucinations, Magnetoencephalography, Auditory cortex, Gamma oscillations, Beta oscillations, Predictive coding

## Abstract

The physiological basis for musical hallucinations (MH) is not understood. One obstacle to understanding has been the lack of a method to manipulate the intensity of hallucination during the course of experiment. Residual inhibition, transient suppression of a phantom percept after the offset of a masking stimulus, has been used in the study of tinnitus. We report here a human subject whose MH were residually inhibited by short periods of music. Magnetoencephalography (MEG) allowed us to examine variation in the underlying oscillatory brain activity in different states. Source-space analysis capable of single-subject inference defined left-lateralised power increases, associated with stronger hallucinations, in the gamma band in left anterior superior temporal gyrus, and in the beta band in motor cortex and posteromedial cortex. The data indicate that these areas form a crucial network in the generation of MH, and are consistent with a model in which MH are generated by persistent reciprocal communication in a predictive coding hierarchy.

## 1 Introduction

Hallucinations are false percepts in the waking state that are not consequences of stimuli in the external environment, and can involve any sensory modality. Musical hallucinations (MH) are a type of auditory hallucination characterized by perception of musical sounds in the absence of any external source of music. Their content is often familiar and can be instrumental, vocal or both. While hallucinations of music can occasionally result from focal brain lesions and psychiatric disorders (Keshavan, David, Steingard, & Lishman, 1992; Warren & Schott, 2006; Saba & Keshavan, 1997) the most common cause is hearing loss in the absence of other pathology (Berrios, 1990). This latter group raises the question of how hearing loss alone can lead to the development of complex MH, which is the focus of this study.

Although a number of case studies involving MH have been reported in the literature (for reviews see Evers, 2006; Evers & Ellger, 2004), there are only a few studies that have investigated the brain bases for MH. In order to determine how the states of a hallucinating brain differ from that of a normal brain, these studies have either compared brain activities in the same subject but in two different sessions (Griffiths, 2000; Kasai, Asada, Yumoto, Takeya, & Matsuda, 1999; Shoyama et al., 2010) often separated by several days, or compared brain activity across different population of subjects, with and without hallucinations (Shinosaki et al., 2003; Vanneste, Song, & De Ridder, 2013). A wide range of cortical and sub-cortical areas, which are inconsistent across studies, have been implicated in MH.

A possible contribution to the lack of converging results amongst previous studies is the absence of a paradigm to measure brain activity associated with MH in individual subjects within a single session. Comparing across sessions may highlight changes in neural activity associated with factors other than hallucination intensity, and comparing across subjects might fail to detect parts of the neural substrate that show inter-individual variation or erroneously imply that certain areas are involved in all subjects. Given the variation in phenomenology in MH, and in subject factors such as musical expertise, the possibility of such inter-individual variation in neuro-anatomical substrate must be seriously considered. We consider here whether a common physiological mechanism might exist that could have different anatomical instantiations to explain the variety of phenomenology and substrates previously reported.

Measuring the brain activity that changes with MH in the same subject in the same session, requires a paradigm in which the MH can be altered at defined times during the course of the experiment. We assessed here whether residual inhibition (RI), which has been successfully used in tinnitus research (Feldmann, 1981; Roberts, 2007; Sedley, Teki, Kumar, Barnes, et al., 2012), might also be applied to MH. RI involves presenting an auditory ‘masker’ stimulus for a period of time, and after this stimulus ends, there is a period of time in which the phantom percept remains reduced in intensity. Contrasting this period of suppressed tinnitus with a nearby period of unsuppressed tinnitus allows measurement of brain correlates of tinnitus in the absence of any external sound stimulation (Kahlbrock & Weisz, 2008; Osaki et al., 2005; Sedley, Teki, Kumar, Barnes, et al., 2012). While the utility of RI in tinnitus is well-established, the same phenomenon has not been reported in MH. The present study focuses on a subject, who is at present unique in the literature, whose MH could be residually inhibited using short periods of music as a masker stimulus. We used whole-head magnetoencephalography (MEG) to contrast oscillatory brain activity during periods of high and low hallucinations. During periods of higher, compared to lower hallucination intensity, we found increases in band-limited oscillatory activity in a left-lateralised network of brain regions.

## 2 Materials and methods

### 2.1 Subject

**Fig. 1:**
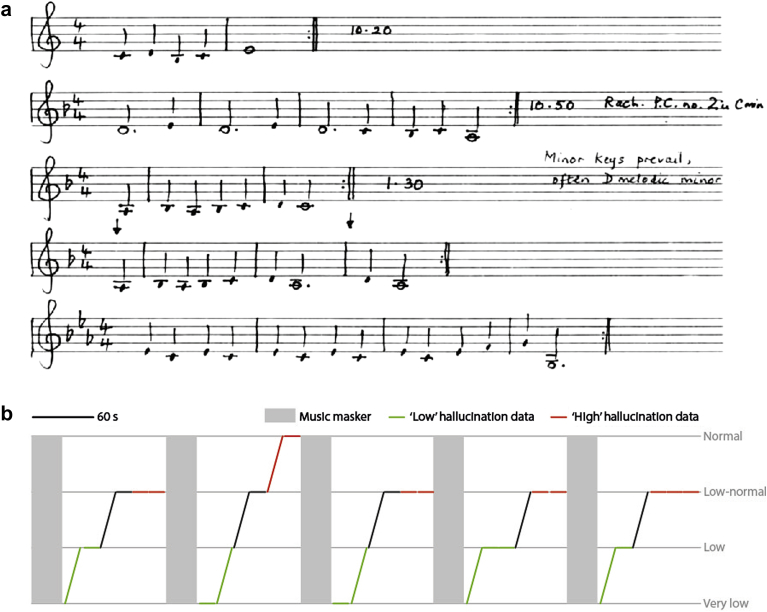
Phenomenology of the subject's musical hallucinations. **a**: Musical notation, made by the subject, of hallucinations experienced on a typical day. Sequences of 2–4 bars in length are each repeated for periods of tens of minutes. The subject identified the second sequence as belonging to Rachmaninov's Piano Concerto number 2 in C minor. **b**: Residual inhibition paradigm used during the experiment, along with subjective ratings of hallucination intensity (grey lines and text). Over the course of the experiment (horizontal axis), 5 music maskers were played (grey rectangles) for 30 sec, each followed by 6 blocks of 15 sec of silence, before and after each of which the subject made a rating of her current hallucination intensity. Each block was therefore defined by its preceding and subsequent hallucination ratings, and is represented by a line in the figure. The 22 blocks whose MEG data were used for analysis are indicated by green or red lines, indicating their assignment to the ‘low’ or ‘high’ hallucination condition respectively.

The subject was a 66 year old right handed woman. She was a Maths teacher and keen amateur musician in that she was an accomplished keyboard player and had absolute pitch. Her MH consisted of instrumental (piano) melodies without any vocals. She demonstrated palinacousis: she often experienced music that was similar to melodies she had recently heard. She would hear the hallucinations most of the time but the content and severity of hallucinations varied from day to day. Being a musician allowed her to formally document in musical notation the melodies that she heard. Fig. 1(a) shows examples of her experiences over part of one day. She had no verbal hallucinations and no past history of neurological or psychiatric disorder.

The subject had developed a degree of hearing loss 20 years before testing. Three years before testing she experienced sudden acute bilateral hearing loss. She also developed bilateral tinnitus (in the form of hissing and chimes) at this time and hyperacusis (experiencing sounds comfortable to most people as intolerably loud). Her perception of music was also distorted such that she had difficulty recognising pitch, melody and key. She started using hearing aids at this time, which were of some benefit. She subsequently, with considerable time and effort, retrained herself to recognise pitch, key and melody. Her hearing loss persisted until the time of the study, at which time her most recent pure-tone audiogram showed a relatively flat profile of 50–65 dB HL thresholds in her left ear, and progressively increasing thresholds in the right ear from 32 dB HL at .5 kHz to 85 dB HL at 8 kHz. During the experiment she did not wear hearing aids, or experience tinnitus.

Her MH started 15 months after the acute loss of hearing. She initially thought the music was actually being played outside but came to realise that there was no external musical stimulus. Initially the music consisted of repetition of just two notes, but grew in length and complexity over time into the recognisable melodies of several bars in length that she experienced at the time of the experiment. She regarded the hallucinations as a nuisance, and was only bothered by them when her mood was already low for other reasons.

### 2.2 Stimuli and paradigm

The paradigm used is an adapted RI paradigm as used in tinnitus (Fig. 1(b)), with an external musical masker stimulus presented for 30 sec followed by a period of 90 sec silence during which MEG data were collected that formed the basis of further analysis. The key difference between a classical RI paradigm for tinnitus and our paradigm was that the former typically uses noise or a pure-tone as a masker, while we used short pieces of classical music. We presented excerpts of music by Bach, as maskers, at a sound level chosen by the subject to be comfortable and clearly audible in both ears. These excerpts were selected by the subject as pieces that she had found to suppress her MH. She reported that not all music suppressed her hallucinations, and had not experienced a suppression effect from non-musical sounds. If an RI paradigm is effective then immediately after the offset of masker, hallucinations are reduced in loudness, or eliminated, and subsequently return to normal loudness, typically over tens of seconds. During the post-masker period, the patient rated the severity of hallucination every 15 sec on a 7 point scale from −3 (very low) to 3 (very high) by pressing a key on a keypad. The rating of 0 at the midpoint of the scale corresponds to the typical intensity of the hallucinations she had experienced on that day, prior to testing. To avoid any confounds related to motor processing, the 15 sec period to be used for subsequent analysis was measured from the offset of previous rating period to the onset of next rating (i.e., it did not include any time where she was preparing or executing a motor response). The choice of experimental parameters (duration of masker, silent period and number of ratings during silent period) was based on a psychophysics session before the MEG session. The aim of that session was to select the most effective stimuli that cause RI and to adjust the parameters of these to achieve maximal variation in the hallucination strength during the silent periods.

### 2.3 MEG data collection

MEG data were acquired using a whole-head CTF system with 275 third-order gradiometer channels at a sampling rate of 600 Hz. The data from one channel was discarded due to large artefacts. The position of the head relative to the sensors was continuously localized using three coils (nasion, left and right pre-auricular points), and did not exceed 5 mm during the experiment. The auditory stimuli were presented diotically via a pneumatic system with etymotic earmolds. A total of five blocks were recorded, each consisting of 30 sec of stimulus followed by 90 sec of silence (plus the total time taken to give the ratings of hallucination intensity). In each block, the subject rated the severity of the hallucination every 15 sec following the stimulus offset. Because this 15 sec period was during the inter-rating period, activity in this period was not confounded by motor preparation or response. Subjective ratings of hallucination intensity were made using a three-button box; a rating of hallucination intensity was displayed on the screen (defaulting to 0: ‘Normal’), the left button decreased the rating, the right button increased the rating and the middle button confirmed the rating. Thus every rating was registered with a single press of the middle button (with the right middle finger), thereby eliminating differences in motor activity or planning as a potential confound of MEG results.

### 2.4 MEG data preprocessing

Data analysis was carried out using SPM8 (www.fil.ion.ucl.ac.uk/spm/software/spm8). Data recorded continuously after each of the musical maskers were divided into six 15-sec epochs. Fig. 1(b) shows that the RI was successful in suppressing the intensity of the hallucination in that the epochs immediately after each masker increased toward the typical intensity. Each epoch was defined as a high- or low-intensity epoch based on the subjects' ratings of intensity at the beginning and end of the epoch (with 0 being usual intensity, negative numbers lower intensity and positive numbers higher intensity than usual). Epochs in which the rating remained at or below −2 were defined as ‘low’ intensity and epochs where the rating remained at or above −1 were defined as ‘high’ intensity. The data in the epochs did not include time periods which correspond to the button presses. We used an equal number of trials per condition to prevent biasing in favour of one condition or the other in the determination of threshold based on non-parametric (permutation-based) statistical analysis. As there were only 11 low epochs but 14 high epochs, 3 of the epochs were randomly removed from the ‘high’ hallucination condition.

### 2.5 Beamforming analysis

Beamformers are data-driven spatial filters that project sensor activity to specified source locations in the brain using a linearly weighted sum of the sensor signals. Source power from a given location is reconstructed with unit gain while interference from other brain and non-brain sources is maximally suppressed. We used the linearly constrained minimum variance (LCMV) beamformer (Van Veen, van Drongelen, Yuchtman, & Suzuki, 1997) to localize the sources of activity in different frequency bands. We determined, at each location of the brain in a 10 mm-spaced 3D grid, oscillatory power in three frequency bands: 1–4 Hz (delta), 5–14 Hz (theta/alpha), 14–30 Hz (beta), 30–60 Hz (gamma) and 70–140 Hz (high gamma). For each frequency band, a pseudo-T score was calculated, representing the power during high hallucinations versus the power during low hallucinations, at each brain location. The significance threshold for these scores was calculated using permutation testing (randomly interchanging conditions 1000 times) to get a null distribution of the *t*-statistic. We used this null distribution to set corrected (over the whole-brain volume) thresholds at *p* < .05. Clusters of significant power difference between conditions were displayed on a standard T1 weighted template brain MRI.

## 3 Results

### 3.1 Behavioural results

The experiment was conducted on a subject with MH in the context of hearing loss who had typical phenomenology for this group. She did not experience tinnitus during the experiment. Unusually for this group, this particular subject was musically sophisticated and able to transcribe her experiences. Fig. 1(a) demonstrates a page of her notebook. During the experimental session, the subject reported that she persistently heard MH, in the form of short sequences from the score of Gilbert and Sullivan's musical HMS Pinafore. Whilst the musical maskers (excerpts of music by Bach) were playing she focussed on these and described no imagery or recall of the masker at other times. Immediately following the musical maskers, she returned to experiencing hallucinations of music from HMS Pinafore. There were no arm or hand movements except to perform the button presses required by the experiment. Fig. 1(b) shows the subjective ratings of hallucination intensity throughout the experiment, which were lowest immediately after the masking music and gradually increased toward normal prior to the start of the next masking music. The figure also shows specific blocks of MEG data that form the basis of further analysis. These results provide proof of principle for the use of RI to study MH, though it is not clear at present the proportion of the MH population in whom RI can be achieved.

### 3.2 Sources of oscillatory power change

Four brain regions, all left-lateralised, showed increase in oscillatory activity during higher hallucination intensity compared to low hallucination intensity. Significant power changes, after whole-brain correction, were observed in the theta/alpha, beta and gamma bands, but not the delta or high gamma bands. The anatomical areas showing power changes are discussed below, according to the frequency band of the power change. No equivalent power changes were noted in the right hemisphere, even after dramatically relaxing statistical thresholds. Notably, no areas showed significant decreases in oscillatory power.

### 3.3 Gamma oscillations – anterior superior temporal gyrus

**Fig. 2:**
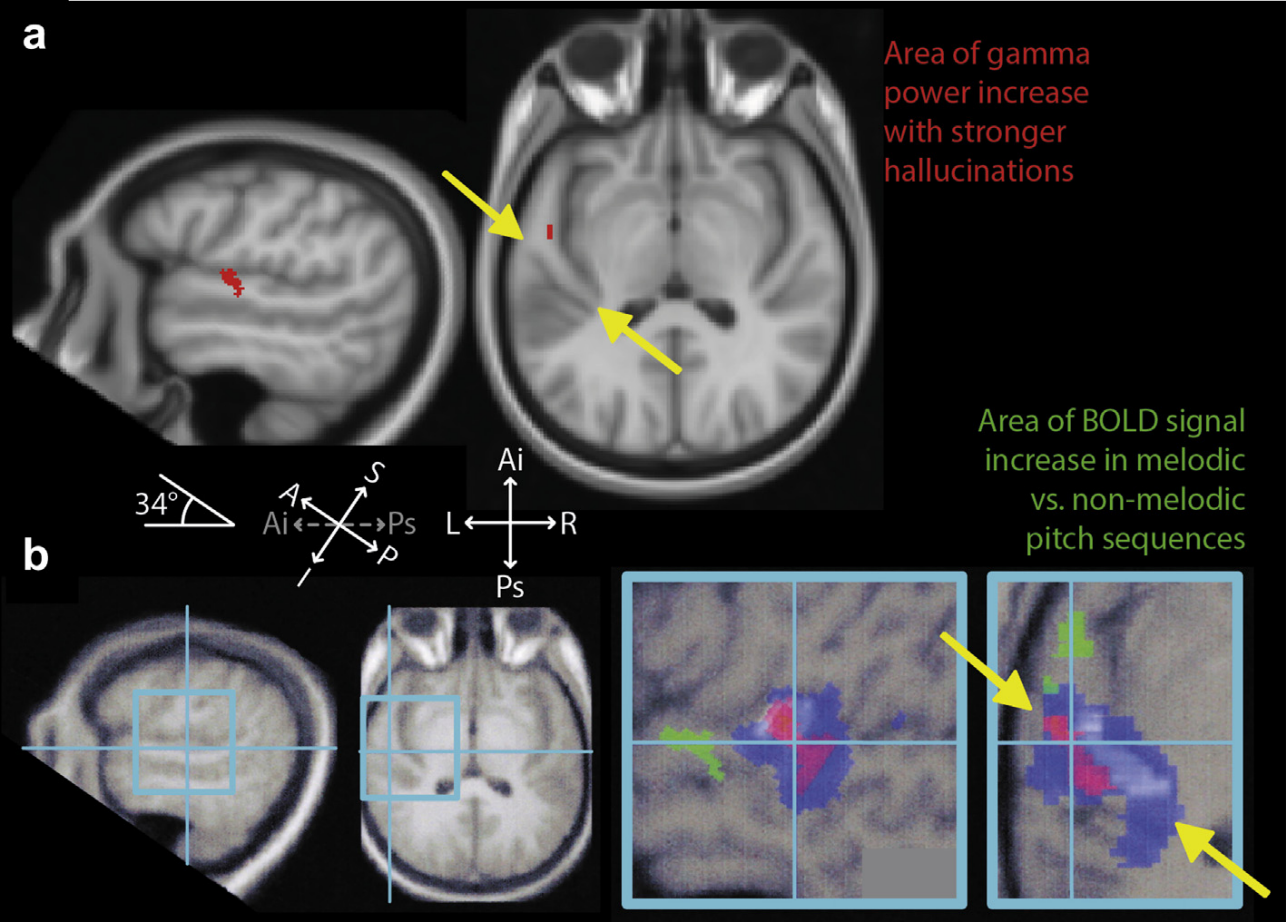
Gamma band (30–60 Hz) oscillatory power increases, in a cortical area specialised for processing pitch sequences (anterior superior temporal gyrus; aSTG), associated with increased (high *vs* low) hallucination intensity. Heschl's gyrus, containing core auditory cortex, runs from posteromedial to anterolateral (ends denoted by yellow arrows), and aSTG is located anterior to its anterolateral end. **a**: Areas of significant gamma power increase surviving whole-brain correction (red areas) displayed on saggital (left) and axial (middle) sections, of a standard template MRI scan, with a 34° tilt applied. **b**: For comparison purposes, the results from a single typical subject from (Patterson et al., 2002) are shown (right) in equivalent tilted saggital and axial sections. The two plots on the left show the positions in the brain of the two enlarged regions on the right. The area responding selectively to melody is shown in green, falling precisely within aSTG, while blue and red areas indicate areas responding to noise and to the pitch of single notes respectively related to Heschl's Gyrus (shown in white). **Abbreviations**: S = superior, I = inferior, A = anterior, P = posterior, Ai = antero-inferior, Ps = postero-superior.

An area of power increase in the gamma band (30–60 Hz) was found in the left anterior superior temporal gyrus [aSTG; MNI co-ordinates (−52 −11 −3)]. Fig. 2(a) illustrates this area of gamma power change while Fig. 2(b) reproduces data from (Patterson, Uppenkamp, Johnsrude, & Griffiths, 2002) to illustrate the position of the area implicated in the perception of melody in a single typical subject.

### 3.4 Beta oscillations – motor cortex and posteromedial cortex

**Fig. 3:**
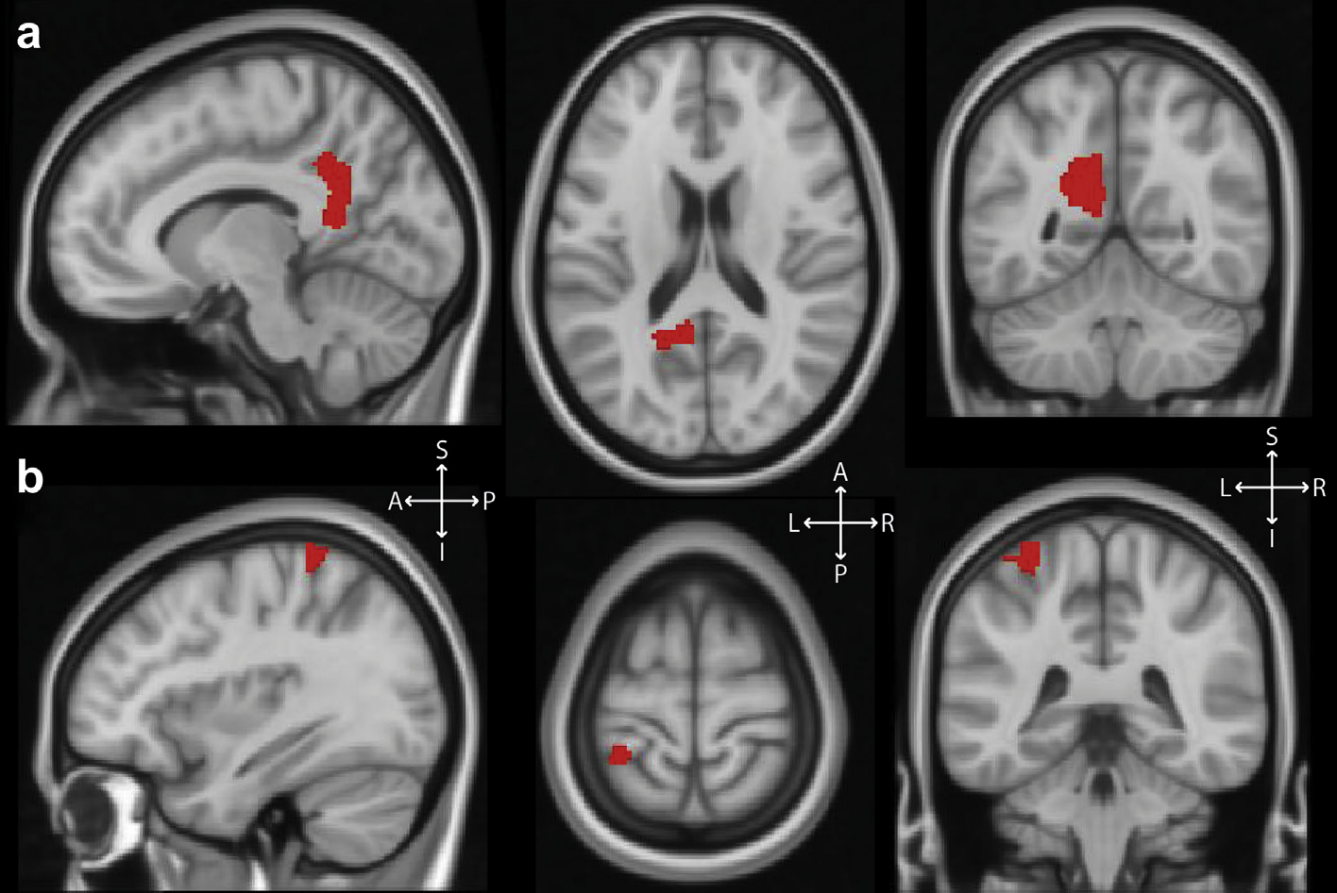
Beta band (14–30 Hz) oscillatory power increases (red areas) associated with increased (high *vs* low) hallucination intensity, displayed on saggital (left), axial (middle) and coronal (right) sections of a standard template MRI scan. **a**: Left posteromedial cortex, comprising a combination of posterior cingulate cortex, precuneus and retrosplenial cortex. **b**: Left primary motor cortex corresponding to the right arm/hand area. **Abbreviations**: S = superior, I = inferior, A = anterior, P = posterior, L = left, R = right.

Power increases in the beta band (14–30 Hz) were found in the left motor cortex [MC; MNI co-ordinates (−30 −41 72)] and the left posteromedial cortex [PMC; MNI co-ordinates (−9 −51 11)], encompassing parts of the posterior cingulate cortex, precuneus and retrosplenial cortex. These areas are shown in Fig. 3.

### 3.5 Theta/alpha oscillations – lateral orbitofrontal cortex (OFC)

In a combined theta and alpha band (5–14 Hz), power increases were seen in the left lateral OFC (−26 48 −14).

### 3.6 Power changes in response to musical stimulation

To see how the observed power changes during hallucinated music compared to those during externally-presented music, we used equivalent beamforming analysis to contrast brain activity during the presentation of the musical maskers to brain activity during baseline states (we used both the pre-music brain activity and post-music brain activity, corresponding to relatively higher and lower hallucination intensities, as alternative baselines). No significant oscillatory power changes were seen in the auditory cortex after whole-brain correction.

## 4 Discussion

In the current work we describe a unique patient whose MH could be successfully manipulated using a RI paradigm. This allowed us to determine, using MEG, changes in the neural activity as a function of intensity of MH. Source-space analysis of the data showed activity in the aSTG (gamma band), MC and PMC (beta band) and OFC (theta/alpha band).

The part of the auditory cortex (aSTG) that shows higher activity during MH coincides with an area implicated in the normal perception of melody (Griffiths, Buchel, Frackowiak, & Patterson, 1998; Patterson et al., 2002) using fMRI. Unfortunately we were unable to directly compare brain activity corresponding to externally-presented and hallucinated music in this patient, as the former did not produce significant changes in oscillatory power. Previous intracranial and MEG studies show that these stimuli should be associated with high-frequency gamma responses (mainly 80 Hz upwards) (Griffiths et al., 2010; Millman, Prendergast, Hymers, & Green, 2013; Nourski et al., 2009; Sedley, Teki, Kumar, Overath, et al., 2012) but the signal-to-noise ratio of these specific gamma power changes with MEG is extremely low, with successful detection using MEG requiring very large numbers of stimuli and group-level analyses (Millman et al., 2013; Sedley, Teki, Kumar, Overath, et al., 2012). It is not yet understood why phantom percepts are associated with much stronger gamma oscillations, as measured with MEG and electroencephalography (EEG), than those associated with external sensory stimulation; for review see (Sedley & Cunningham, 2013).

The motor system has been shown to be active even during passive listening to music (Chen, Penhune, & Zatorre, 2008) and during musical imagery in musicians (Haueisen & Knösche, 2001; Meister et al., 2004). This activity, therefore, likely reflects involvement of motor areas in musical imagery processes associated with the generation of hallucinations.

Posteromedial cortex forms part of the default mode network (Buckner, Andrews-Hanna, & Schacter, 2008; Raichle et al., 2001) within which the retrosplenial cortex, has been suggested to have a specific role in the representation of permanent landmarks (such as objects in a virtual 3D landscape that are always present in a given location) (Auger, Mullally, & Maguire, 2012). MH can be considered a permanent ‘landmark’ in the auditory scene once the music has been present for a certain length of time. Increased activity also occurs in PMC during retrieval of auditory memories (Buckner, Raichle, Miezin, & Petersen, 1996; Huijbers et al., 2012), auditory imagery (Seung-Schick, Uk, & Gil, 2001) and the perception of unpleasant music (Blood, Zatorre, Bermudez, & Evans, 1999). Taken together, these observations suggest that PMC has several roles in perception and memory, particularly with regard to pervasive objects. In MH, these may relate to the retrieval of musical melody from memory, and generation of the musical imagery.

We obtained higher activity in the alpha band in the OFC during periods of MH. The OFC is known to be involved in the representation and assignment of emotional valence to stimuli (Rolls, 2007) and is shown to be active in response to unpleasant music (Blood et al., 1999). It is, therefore, unsurprising that its activity was found to correlate with the intensity of hallucinations, which were experienced as bothersome to a degree by the subject. This is also consistent with a recent study (Joos, Vanneste, & De Ridder, 2012) which showed activity in the alpha band in OFC correlated with distress caused by the phantom percept of tinnitus. We are not aware of any established or proposed role of this area that would make it a candidate for actually generating the hallucinatory music. However, the activity of this area could potentially both modulate and be modulated by the intensity of hallucinations, serving as a mechanism by which the attribution of emotional salience to MH could further amplify the strength of the percept.

It is interesting to compare our results to a recent study (Vanneste et al., 2013), which used EEG to compare spontaneous activity (SA) in a group of subjects with MH to a group with tinnitus and a group of healthy controls. Although our study and the previous study used different designs (within-subjects *vs* between-subjects, and high/low hallucination contrast *vs* SA), there are some interesting points of convergence. By comparing SA associated with MH to SA associated with the simple phantom percept of tinnitus, Vanneste et al. (2013) showed increased gamma power in the anterior superior temporal plane, which is potentially consistent with our observation of increased gamma power in aSTG (although the hemisphere involved is different, which may reflect the musical expertise of our subject, see below). Similarly, consistent with our study, Vanneste et al. (2013) showed increased power in alpha and beta bands in ‘higher’ areas, which is consistent with the hierarchical model of musical hallucination (see Section 4.3). While the locations of some of these ‘higher’ areas are different in Vanneste et al. (2013) (perhaps due to the heterogeneous neuro-anatomical bases of MH), notably they found strong beta power increases in PMC associated with both MH and simple phantom perception. Another finding of Vanneste et al. (2013) is that both simple and complex phantom auditory percepts were associated with increased gamma power in primary auditory cortex bilaterally. This finding has also been reported in studies of tinnitus (van der Loo et al., 2009; Sedley, Teki, Kumar, Barnes, et al., 2012; Weisz, Wienbruch, Dohrmann, & Elbert, 2005). With these findings in mind, we cannot exclude the possibility our subject had persistently elevated gamma oscillations in primary auditory cortex, but that these simply did not change in magnitude between high and low hallucination states.

Change in power of oscillatory activity in different frequency bands has also been observed in a number of auditory/visual illusions: increase in both beta and gamma bands (Vinnik, Itskov, & Balaban, 2012; Wu & Zhang, 2009) and gamma band alone (Bhattacharya, Shams, & Shimojo, 2002; Kaiser, Bühler, & Lutzenberger, 2004, 2006; Lange, Oostenveld, & Fries, 2013). The results of these studies are consistent with ours in that they point to the role of an increase in beta and gamma oscillations in non-veridical percepts.

### 4.1 Left lateralisation of neural correlates of MH

The left lateralisation of the activity in the auditory cortex leads us to speculate whether the musical expertise of the subject is relevant. Data on left lateralized mechanisms for auditory analysis by musicians include behavioural (Bever and Chiarello, 1974; Johnson, 1977), structural (Schlaug, Jancke, Huang, & Steinmetz, 1995) and functional (Ohnishi et al., 2001) brain imaging. The asymmetry of hearing loss in this patient might also be relevant. This subject had greater low-frequency hearing loss in the left ear which is in a spectral region that is important to music and might have led to asymmetry of musical stimulation in favour of the left hemisphere and a smaller masking effect in the right hemisphere.

### 4.2 Evidence for hierarchical communication

A number of studies have demonstrated schemes in which gamma activity occurs in hierarchically lower areas and beta frequency activity in areas that are higher in the cortical hierarchy (Arnal, Wyart, & Giraud, 2011; Bastos et al., 2012; Wang, 2010). In such schemes, ascending communication from the lower to the higher areas occurs in the high-frequency (gamma) signal and descending communication from the higher to the lower areas in the low-frequency (beta) signal. Interpreted in the light of such models, our data suggest that MH arise from hierarchical communication between aSTG (lower in the hierarchy) and both PMC and MC (higher in the hierarchy). The roles of these cortical areas in normal musical cognition also suggest hierarchical communication of this type: a hierarchy based on perceptual activity in lower areas and activity related to imagery and memory in higher areas.

### 4.3 A canonical model of MH

Here we present a new model to explain the development and maintenance of MH. This is based entirely on known neural processes, and requires no pathology other than hearing loss in order for MH to develop. Our present MEG findings are in keeping with this model.

#### 4.3.1 Predisposing factors for developing MH

MH is a rare phenomenon: although estimates vary across studies, less than one percent of population who have acquired hearing loss develop MH (Cope & Baguley, 2009). We first consider why some people but not others with acquired hearing loss develop complex hallucinations of music and why complex auditory hallucinations in acquired hearing loss preferentially take the form of music rather than other percepts such as speech or environmental sounds.

##### 4.3.1.1 Characteristics of the individual

In our model, top-down predictions (or ‘priors’) in the auditory system are crucial in the development of MH. These are influenced by previous experience, beliefs and expectations. Musical exposure and the importance attached to music by the individual might therefore be relevant. This is relevant to the subject of the current study who is a keen and accomplished amateur musician. But MH can also develop in the absence of any musical training. Studies of the factors that influence spontaneous music imagery (a ‘tune stuck in the head’ or ‘ear worm’) may be informative here, as the normal substrates for musical perception and spontaneous music imagery show considerable overlap (Kraemer, Macrae, Green, & Kelley, 2005) and a mechanism for ear worms might be based on similar neural architecture and physiology to the one we are proposing for MH. A positive correlation between the frequency and duration of ear worms and musical skill (Bailes, 2007; Liikkanen, 2008, 2011) has been demonstrated. Beaman and Williams (2010) further showed that it is not the musical skills *per se* but the ‘subjective importance’ attached to music that predicted the frequency of ear worms. This might explain why people with no musical skills but who nevertheless regard music as an important part of their life (e.g., listening to music for entertainment) can also get MH. The possible relevance of abnormalities of attention (Collerton, Perry, & McKeith, 2005) as a predisposing factor also merits further investigation.

##### 4.3.1.2 Characteristics of music

While complex hallucinations following acquired hearing loss in the form of music are well-described (see references in the Introduction), reports of other types of complex auditory hallucination (such as voices) that are uniquely associated with hearing loss, in the absence of other factors, are rare. Sommer et al. (2010) describe verbal hallucination in some normal individuals, but the hallucinations were associated with factors that are not uniquely attributable to the hearing loss. We have encountered a very small number of patients with MH who also experience verbal hallucinations without evidence of psychotic illness (personal observation TD Griffiths: two patients in a series of fifty). This suggests that such patients do exist, but with a low prevalence.

We propose that patients with hearing loss experience musical rather than other types of hallucination because of the statistical properties of music. Music, compared to speech and language, is more *predictable* (Fitch, 2006) and *repetitive* (see Introduction chapter in Ockelford, 2005 and references therein). Predictable means that hearing the present note, or few notes, is sufficient to predict the upcoming notes, either by its mathematical rules (Voss & Claske, 1975; Levitin, Chordia, & Menon, 2012) or by retrieval from memory (Schellenberg, Iverson, & McKinnon, 1999). The predictability of music may be due to discretization of both pitch (scale) and temporal (beat) dimensions in music, in contrast to speech where both dimensions are continuous (Fitch, 2006). Repetitive means that a given segment of music (a bar or a melody) is repeated over a course of time. Repetition in music is shown to be important for its emotional and aesthetic value (Garcia, 2005; Pereira et al., 2011) and is such a critical aspect for distinguishing it from speech that if a spoken sentence is repeated several times over, it starts sounding like music (Deutsch, Henthorn, & Lapidis, 2011). Because of these properties of music, the percept of music once initiated is selectively reinforced to persist because of the repetitiveness and predictability of music. Moreover, since violations of predictions/expectations of music percept evoke negative emotions (Steinbeis, Koelsch, & Sloboda, 2006), the percept is continued as per expectations. We argue that it is this recursive cycle that is uniquely applicable to music which may explain why the content of hallucinations following hearing loss is predominantly music. However we do not argue that non-musical complex percepts (such as speech) *cannot* occur in acquired hearing loss, but rather that the properties of music mentioned above make it much more likely to be the subject of hallucinations following hearing loss.

#### 4.3.2 Neuronal model of MH

Our model of MH is based on the ‘predictive coding’ theory of brain function (Bastos et al., 2012; Kumar et al., 2011; Rao & Ballard, 1999). In this framework, each level of the cortical hierarchy tries to predict the representation of sensory objects in the level below by sending top-down predictions. Aspects of the representation that are inconsistent with the prediction (the *prediction error*) are then passed back to the higher level. Prediction errors are then used to update the representations at the higher level. In this framework, all bottom-up (ascending) connections communicate prediction error, and top-down (descending) connections convey predictions. This message passing changes hierarchical representations such that prediction error is minimized at all levels. In this regard, the predictive coding framework is Bayes-optimal from the perceptual inference perspective (Friston, 2010).

**Fig. 4:**
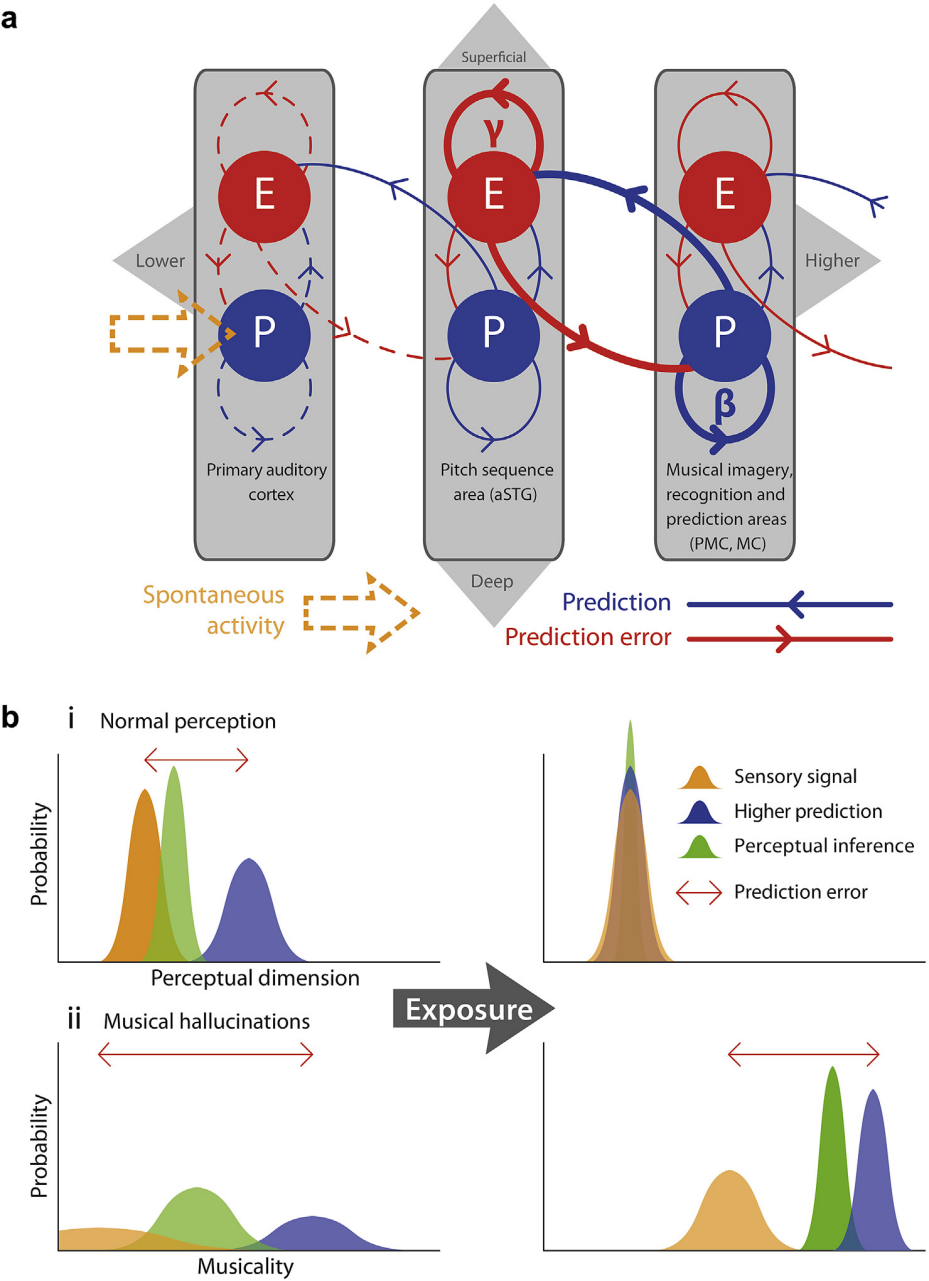
Predictive coding model of musical hallucinations. (**a**) Neural architecture of proposed model. Three levels of a cortical hierarchy for music processing are depicted (primary auditory cortex, aSTG and PMC/MC in order of lower to higher). Each cortical area comprises prediction error (E) populations in the superficial layers which oscillate at gamma frequencies, and prediction (P) populations in the deep cortical layers which oscillate at beta frequencies. Bi-directional communication occurs between P and E populations within each level and between each pair of adjacent levels. Thicker lines represent more precise predictions and predictions errors, which constitute the fundamental hallucinatory circuit, while dashed lines represent imprecise activity driven by spontaneous noise-like input from sub-cortical pathways. aSTG = anterior superior temporal gyrus. PMC = posteromedial cortex. MC = motor cortex. (**b**) Schematic of Bayesian inference (**i**) normal perception. The left panel illustrates the state of the system at stimulus onset, with a relatively precise sensory signal, a less precise prediction and a prediction error due to incongruence between these. The right panel illustrates the system after a short interval (∼100 msec), during which the higher prediction has been modified to become congruent with the sensory signal and more precise. The perceptual inference is therefore veridical (**ii**) Bayesian inference in musical hallucinations. The left panel shows the state of the system when hallucinations are low in intensity. Imprecise SA with relatively high precision top-down prediction is combined to infer a weak musical percept. After reinforcement, the top-down prediction becomes more precise (right panel) and therefore a strong percept of music (hallucinations) is inferred.

A schematic representation of the predictive coding – using three levels of a hierarchy – is shown in Fig. 4(a). Each level comprises two neuronal populations marked ‘P’ (prediction) and ‘E’ (error). Prediction populations are located in deep cortical layers, while error populations are located superficially. These populations are reciprocally connected within their levels and with the next hierarchical level, such that each prediction population updates its prediction based on prediction error from its own level and the level below. Conversely, each error population encodes its prediction error based on predictions from its own level and the level above. Evidence from electromagnetic recordings in humans (Arnal et al., 2011; Iversen, Repp, & Patel, 2009) suggest that top-down prediction and bottom-up prediction error may use different frequency bands; where descending predictions are transmitted in the beta range (blue arrows in the figure) and ascending prediction errors are conveyed predominantly in the gamma band (red arrows in the figure). See (Bastos et al., 2012) for a fuller discussion.

A crucial aspect of this model is the relative contribution of bottom-up (prediction error) and top-down (prediction) influences on representational updates. Perceptual inference – at a given level of the hierarchy – rests on the influence of prediction errors from lower levels, relative to the prediction error at the level in question. In predictive coding, these influences are proportional to ‘precision’, which is an estimate of the signal-to-noise ratio or reliability of the prediction error (Feldman & Friston, 2010). Physiologically, precision is thought to be encoded by the post-synaptic gain of the neurons that encode prediction error; namely, superficial pyramidal cells (Bastos et al., 2012; Mumford, 1992). If the gain of superficial pyramidal cells is relatively high in sensory areas, the propagation of sensory input (sensory prediction error) up the hierarchy is facilitated and top-down predictions are changed to match sensory input. In this context, the percept is dominated by sensory input. On the other hand, if post-synaptic gain is relatively higher in upper levels, then top-down predictions are more precise and will dominate perceptual inference – being relatively impervious to imprecise bottom-up influences.

Generalised models of predictive coding (Feldman & Friston, 2010) suggest that the precisions at different hierarchical levels depend on the context (e.g., paying attention to a particular feature of the sensory stimulus will increase the precision of pathways reporting that feature). In these models, precisions are estimated in much the same way the causes of sensory input. Specifically, the top-down input not only predicts the input at the lower level (content) but also predicts the precision (context) at that level. The important point here is that the precision or post-synaptic gain, at a given level, can be adjusted by a top-down input. Mechanistically, post-synaptic gain can be changed by several factors that include fast oscillatory activity (Fries, Womelsdorf, Oostenveld, & Desimone, 2008) and the activity of neuromodulators such as acetylcholine (Yu & Dayan, 2005).

In the current context, we can consider three levels of the cortical hierarchy that comprise primary auditory cortex (A1) at the lowest, aSTG at the middle and PMC/MC at highest level. Primary auditory cortex is the gateway to auditory cortex for all acoustic stimuli and is therefore lowest in the hierarchy. The aSTG is hierarchically below the PMC/MC because, while aSTG has been shown to be involved in sensory perception of music (Patterson et al., 2002), PMC/MC are involved in high level cognitive tasks related to music. For example, PMC is involved in imagery and retrieval of episodic memories for music (Halpern & Zatorre, 1999; Janata, 2009) and is thought to be involved in storage of amodal conceptual knowledge (Fairhall & Caramazza, 2013). Similarly, MC is involved in musical imagery, especially in musicians (Haueisen & Knösche, 2001; Meister et al., 2004) and is known to modulate – in a top-down fashion – auditory cortex (Iversen et al., 2009). The observed frequency bands of power change in our data (gamma in aSTG and beta in PMC/MC) further support this hierarchical interpretation.

Crucially, we consider MH to be the result of aberrant hierarchical precision or gain control that results from hearing loss. In animal models of hearing loss, recordings from animals show SA in almost all centres of the auditory hierarchy (including the dorsal cochlear nucleus, inferior colliculus and primary and secondary auditory cortex (see Kaltenbach, 2011 for review). Studies investigating evolution of the time course of SA show that elevated SA in the central auditory system is a consequence of passive relay of activity from the lower auditory centres (Manzoor, Gao, Licari, & Kaltenbach, 2013; Mulders & Robertson, 2009, 2011). The increased SA in lower auditory centres could be because of local homeostatic mechanisms (Turrigiano, 2008; for review see Noreña, 2011) to restore the baseline activity following hearing loss.

Our hypothesis is that peripheral hearing loss reduces the signal-to-noise ratio of incoming auditory stimuli and the brain responds by decreasing sensory precision or post-synaptic gain. In our model, this happens at or below the level of primary auditory cortex. Because of relatively higher (compared to A1) precision, aSTG conveys relatively precise prediction error to PMC/MC (in the gamma band) and PMC/MC reciprocates predictions to aSTG (in the beta band). A recurrent loop of communication is thus established between aSTG and PMC/MC which is no longer informed, or entrained, by precise bottom-up sensory prediction errors. Spontaneous activities in these areas, therefore, correspond to autonomous perceptual predictions. Since the precision of ascending sensory information is low, top-down predictions in this recurrent loop are only constrained by a need to preserve the internal consistency between hierarchial representations of music in aSTG and PMC/MC. This reciprocal communication between an area in music perception (aSTG) and area/s involved in higher music cognition (PMC/MC) with no constraint from the sensory input gives rise to MH. In summary, it is the adaptive reduction of sensory precision (estimated signal-to-noise ratio) that permits the emergence of hallucinatory predictions or percepts that are inferred with a relatively high degree of precision or confidence. A heuristic illustration of this perceptual inference during normal perception and during MH is shown in Fig. 4(b).

The auditory systems of all people with a sufficient degree of acquired hearing loss presumably undergo these adaptive changes in relative precision, yet only a small minority of these individuals develop MH. We propose that the critical step in developing MH, in the context of reduced sensory precision, is the establishment of predictions and prediction errors, consistent with music, that are strong enough to result in the recursive cycle of self-reinforcement described above. Reaching this state is most likely the combination of characteristics of the individual, as described above and chance combinations of internal and external circumstances.

It should be noted that our model is not tied to specific anatomical locations but argues for a specific and aberrant pattern of communication between ‘higher’ and sensory levels within the hierarchical framework of predictive coding. Given the empirical and theoretical evidence that ascending prediction errors and descending predictions are conveyed in distinct frequency bands, the pattern of changes in reciprocal message passing could be tested empirically using inter-frequency causal interactions (Chen, Kiebel, et al., 2008; Moran et al., 2009) or the direct estimation of post-synaptic gain, using dynamic causal modelling (Moran et al., 2013).

#### 4.3.3 Explaining RI in terms of predictive coding

As observed in the current study, external music reduced the intensity of hallucinations. Furthermore, hallucinations remained low in intensity for almost a minute or so after the offset of music (residual suppression). We now explain this effect using our model.

Under the predictive coding model, hallucinations arise from recurrent interactions between PMC/MC and aSTG that are not constrained by the sensorium because of the attenuation of sensory precision. If a precise sensory input is available, as when listening to music in a high signal-to-noise context, hierarchical perception will be entrained to predict precise sensory input. The spontaneous autonomous dynamics is suppressed, thereby stopping or reducing the intensity of hallucinatory percept. Furthermore, one might anticipate that there would be a transient increase in sensory precision (post-synaptic gain) that reflects the increase in auditory signal-to-noise. If this transient increase persisted for a few minutes (through enduring changes in post-synaptic sensitivity), the emergence of spontaneous autonomous perceptual dynamics would be suppressed temporarily. This suggests that RI of musical hallucinosis should be accompanied by a transient increase in the gain of the sensory areas – a prediction that, in principle, could be tested empirically.

As the current study is the first to report RI in MH, effectiveness of RI in a larger population of subjects needs to be empirically tested in future studies.
