# Supplementary material for: A brain basis for musical hallucinations
Source: Cortex. 2014 Mar;52(100):86–97. doi: 10.1016/j.cortex.2013.12.002 (PMC3969291; doi:10.1016/j.cortex.2013.12.002)
Supplement: Supplementary file 1 [file mmc1.docx]

**Supplementary Material**

**Figure S1: Power Spectrum for the time series from aSTG**

**Figure S2: Power Spectrum for the time series from motor cortex**

**Figure S3: Power Spectrum for the time series from posteromedial cortex**

**

**Figure S4: Power Spectrum for the time series from orbitofrontal cortex**
